# Crystal structure of bis­{3-(3-bromo-4-methoxyphenyl)-5-[6-(1*H*-pyrazol-1-yl)pyridin-2-yl]-1,2,4-triazol-3-ato}­iron(II) methano­l disolvate

**DOI:** 10.1107/S2056989022010179

**Published:** 2022-10-28

**Authors:** Kateryna Znovjyak, Igor O. Fritsky, Tatiana Y. Sliva, Vladimir M. Amirkhanov, Sergey O. Malinkin, Sergiu Shova, Maksym Seredyuk

**Affiliations:** a Department of Chemistry, Taras Shevchenko National University of Kyiv, Volodymyrska Street 64, Kyiv, 01601, Ukraine; b Department of Inorganic Polymers, "Petru Poni" Institute of Macromolecular Chemistry, Romanian Academy of Science, Aleea Grigore Ghica Voda 41-A, Iasi 700487, Romania; Universidad de Los Andes Mérida, Venezuela

**Keywords:** crystal structure, spin-crossover, spin transition, energy frameworks

## Abstract

The unit cell of the title compound, [Fe^II^(C_17_H_12_BrN_6_O)_2_]·2MeOH, consists of a charge-neutral complex mol­ecule and two independent mol­ecules of methanol. In the complex mol­ecule, the two tridentate ligand mol­ecules 2-[5-(3-bromo-4-meth­oxy­phen­yl)-4*H*-1,2,4-triazol-3-yl]-6-(1*H*-pyrazol-1-yl)pyridine coordinate to the Fe^II^ ion through the N atoms of the pyrazole, pyridine and triazole groups, forming a pseudo-octa­hedral coordination sphere around the central ion. In the crystal, neighbouring asymmetric mol­ecules are linked through weak C—H(pz)⋯π(ph) inter­actions into chains, which are then linked into layers by weak C–H⋯N/C inter­actions. Finally, the layers stack into a three-dimensional network linked by weak inter­layer C—H⋯π inter­actions between the meth­oxy groups and the phenyl rings. The inter­molecular contacts were qu­anti­fied using Hirshfeld surface analysis and two-dimensional fingerprint plots, revealing the relative contributions of the contacts to the crystal packing to be H⋯H 34.2%, H⋯C/C⋯H 25.2%, H⋯Br/Br⋯H 13.2%, H⋯N/N⋯H 12.2% and H⋯O/O⋯H 4.0%. The average Fe—N bond distance is 1.949 Å, indicating the low-spin state of the Fe^II^ ion. Energy framework analysis at the HF/3–21 G theory level was performed to qu­antify the inter­action energies in the crystal structure.

## Abstract 2

The title charge-neutral complex is a low-spin complex with a moderately distorted pseudo-octa­hedral coordination environment of the metal ion. As a result of their asymmetric shape, the mol­ecules stack into chains, which eventually pack into layers and, finally, into a three-dimensional network connected by weak C—H⋯N, C—H⋯C hydrogen bonds and C—H⋯π inter­actions.

## 1. Chemical context

A broad class of coordination compounds exhibiting spin-state switching between low- (total spin *S* = 0) and high-spin states (total spin *S* = 2) is represented by Fe^II^ complexes based on tridentate bis­azole­pyridine ligands (Halcrow, 2014 ▸; Suryadevara *et al.*, 2022 ▸; Halcrow *et al.*, 2019 ▸). In the case of asymmetric ligand design, where one of the azole groups carries a hydrogen on a nitro­gen heteroatom and acts as a Brønsted acid, deprotonation can produce neutral complexes that can be either high-spin (Schäfer *et al.*, 2013 ▸) or low-spin (Shiga *et al.*, 2019 ▸) or exhibit temperature-induced transitions between the spin states of the central atom (Seredyuk *et al.*, 2014 ▸), depending on the ligand field strength. The periphery of the mol­ecule, *i.e*. ligand substituents, also plays an important role in the behaviour, determining the way in which mol­ecules are packed in the lattice and their inter­actions with each other, and therefore further influencing the spin state adopted by the central atom. As we have recently demonstrated, the dynamic rearrangement of the meth­oxy group between the bent and extended configurations can lead to a highly hysteretic spin transition *via* a supra­molecular blocking mechanism (Seredyuk *et al.*, 2022 ▸). 
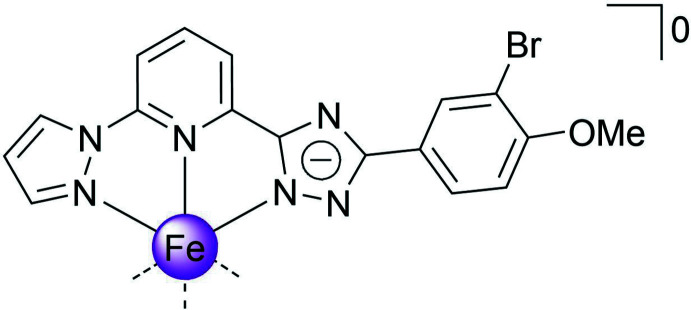


Having inter­est in spin-transition 3*d*-metal complexes formed by polydentate ligands (Bartual-Murgui *et al.*, 2017 ▸; Bonhommeau *et al.*, 2012 ▸; Valverde-Muñoz *et al.*, 2020 ▸), we report here on our current structural exploration of a new complex [Fe^II^ *L*_2_] based on an asymmetric deprotonable ligand with two substituents on the phenyl group, *L* = 2-[5-(3-bromo-4-meth­oxy­phen­yl)-4*H*-1,2,4-triazol-3-yl]-6-(1*H*-pyrazol-1-yl)pyridine.

## 2. Structural commentary

**Figure 1:**
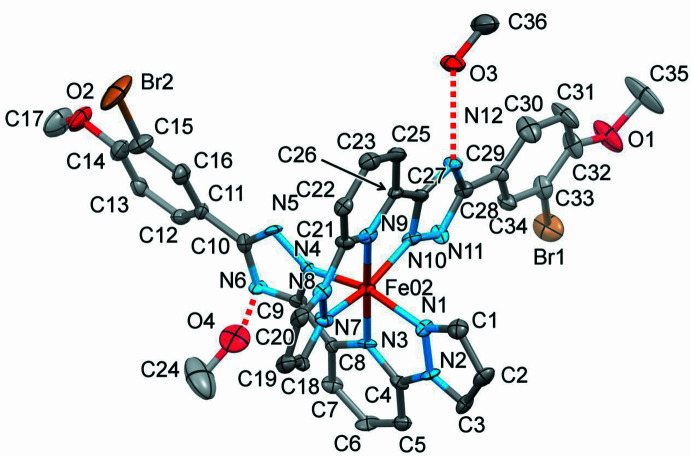
The mol­ecular structure of the title compound with displacement ellipsoids drawn at the 50% probability level. H atoms have been omitted for clarity. Hydrogen bonds are indicated by dashed lines.

The title complex has a asymmetric mol­ecule with divergent phenyl groups. The ligand mol­ecules are almost planar (r.m.s. deviation = 0.330 Å), including the meth­oxy substituents, which also lie in the plane of the aromatic groups [atoms C17 and C35 are 0.514 (1) and 0.116 (1) Å, respectively, away from the planes passing through their respective ligand molecules]. The two independent methanol mol­ecules form O—H⋯N hydrogen bonds with the triazole (trz) rings of the ligand mol­ecules (Fig. 1 ▸, Table 1 ▸). The central Fe^II^ ion of the complex has a distorted octa­hedral N_6_ coordination environment formed by the nitro­gen donor atoms of two tridentate ligands (Fig. 1 ▸).

The average bond length, <Fe—N> = 1.949 Å, is typical for low-spin complexes with an N_6_ coordination environment (Gütlich & Goodwin, 2004 ▸). The average trigonal distortion parameters *Σ* = Σ_1_^12^(|90 − *φ*_i_|), where *φ*_i_ is the angle N–Fe–N′ (Drew *et al.*, 1995 ▸), and *Θ* = Σ_1_^24^(|60 − *θ*_i_|), where *θ*_i_ is the angle generated by superposition of two opposite faces of an octa­hedron (Chang *et al.*, 1990 ▸) are 93.3 and 298.8°, respectively. The values reveal a deviation of the coordination environment from an ideal octa­hedron (where *Σ* = *Θ* = 0) but is, however, in the expected range for bis­azole­pyridines and similar ligands (see below). The calculated continuous shape measure (CShM) value relative to the ideal *O_h_* symmetry is 2.24 (Kershaw Cook *et al.*, 2015 ▸). The volume of the [FeN_6_] coord­ination polyhedron is 9.536 Å^3^.

## 3. Supra­molecular features

**Figure 2:**
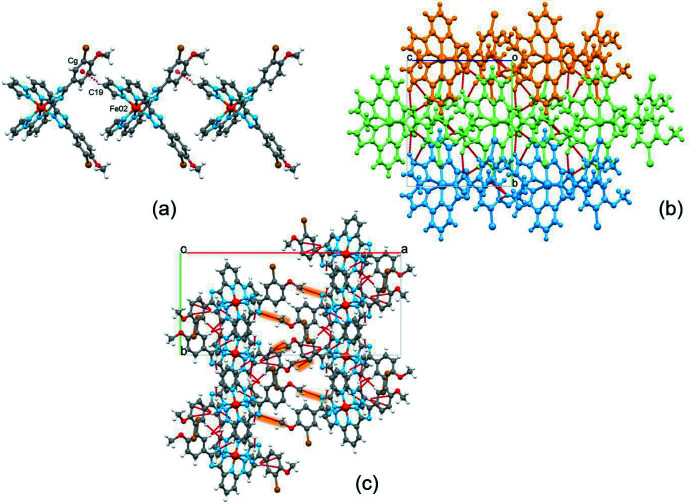
(*a*) Mono-periodic supra­molecular chain formed by stacking of mol­ecules of the title compound. (*b*) Di-periodic layers formed by supra­molecular chains. For a better representation, each chain has a different colour. (*c*) Highlighted inter­actions of neighbouring layers in the three-dimensional supra­molecular network of the title complex. The red dashed lines correspond to contacts below the sum of the van der Waals radii. The methanol mol­ecules are not shown for clarity.

As a result of their asymmetric shape, neighbouring complex mol­ecules fit into each other and inter­act through a weak C—H(pz)⋯π(ph) inter­molecular contact between the pyrazole (pz) and phenyl (ph) groups respectively (Table 1 ▸). The mono-periodic supra­molecular chains formed extend along the *c-*axis direction with a stacking periodicity of 10.6434 (3) Å (equal to cell parameter *c*; Fig. 2 ▸ *a*)*.* Through weak inter­molecular C—H(pz, py)⋯ N/C(pz, trz) inter­actions in the range 3.128 (14)–3.734 (11) Å (Table 1 ▸), neighbouring chains are linked into corrugated layers in the *bc* plane (Fig. 2 ▸ *b*,*c*). The layers stack with inter­layer inter­actions limited to C—H⋯N(trz) and C—H⋯π(ph) contacts involving the methyl groups (Fig. 2 ▸ *c*). The voids between the layers are occupied by methanol mol­ecules, which also participate in bonding between neighbouring layers (see Table 1 ▸ for the complete list of inter­molecular inter­actions).

## 4. Hirshfeld surface and 2D fingerprint plots

**Figure 3:**
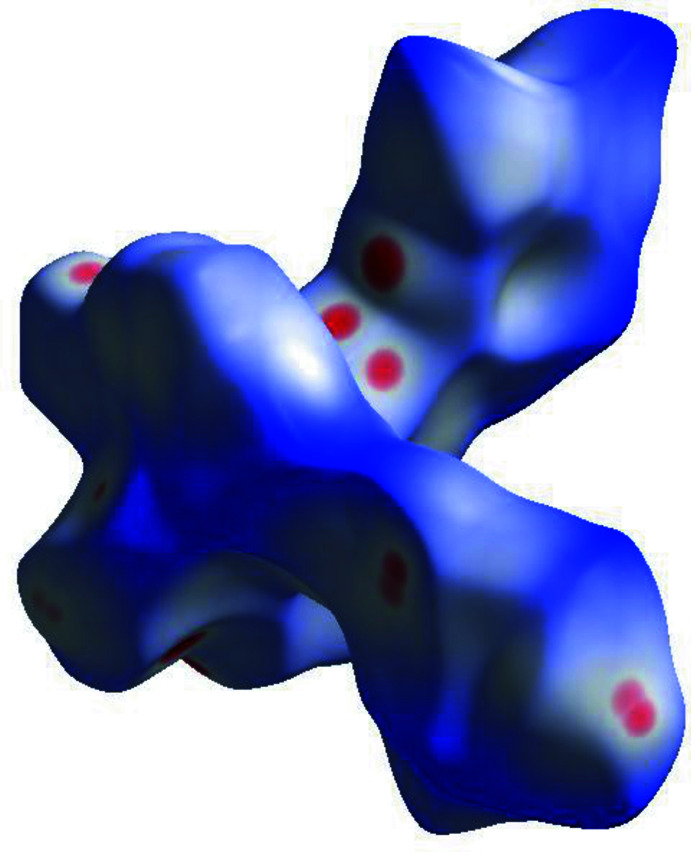
A projection of *d*_norm_ mapped on the Hirshfeld surface, showing the inter­molecular inter­actions within the mol­ecule. Red areas represent regions where contacts are shorter than the sum of the van der Waals radii, blue areas represent regions where contacts are larger than the sum of van der Waals radii, and white areas are regions where contacts are close to the sum of van der Waals radii.

**Figure 4:**
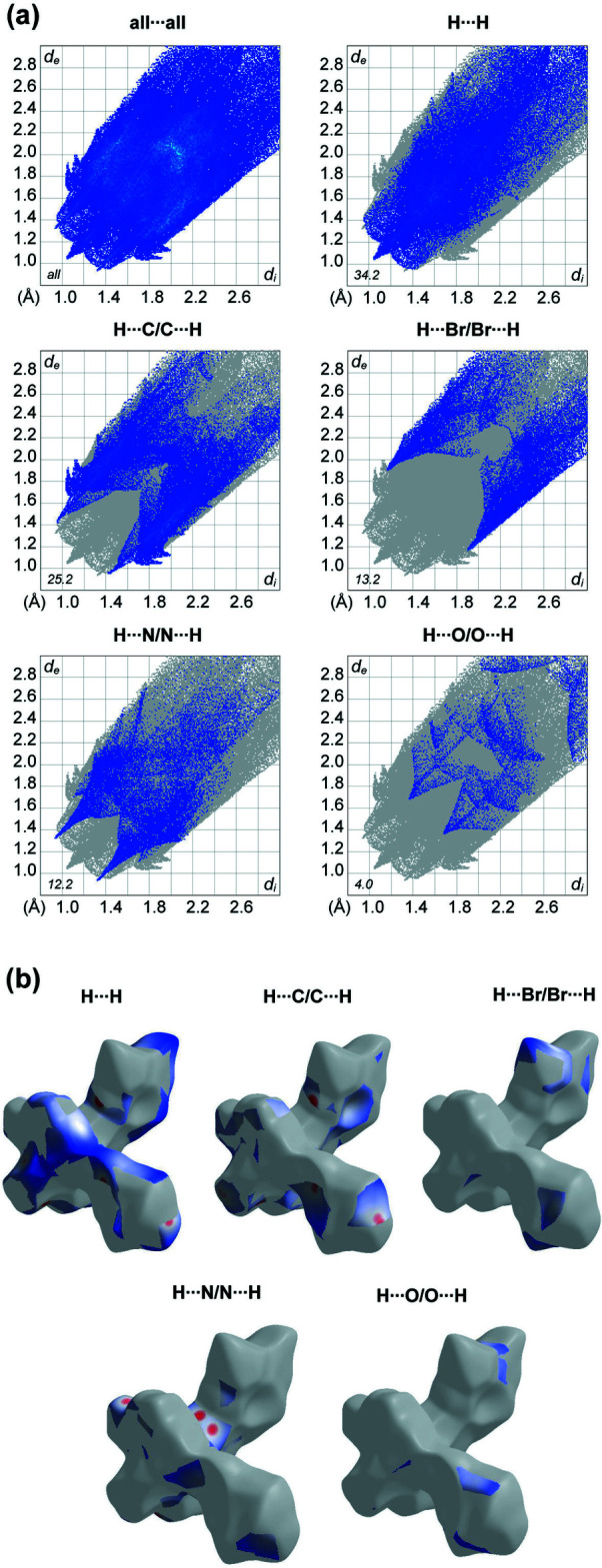
(*a*) The overall two-dimensional fingerprint plot and those decomposed into specified inter­actions. (*b*) Hirshfeld surface representations with the function *d*_norm_ plotted onto the surface for the different inter­actions.

Hirshfeld surface analysis was performed and the associated two-dimensional fingerprint plots were generated using *CrystalExplorer* (Spackman *et al.*, 2021 ▸), with a standard resolution of the three-dimensional *d*_norm_ surfaces plotted over a fixed colour scale of −0.2869 (red) to 2.4335 (blue) a.u. (Fig. 3 ▸). The pale-red spots represent short contacts and negative *d*_norm_ values on the surface corresponding to the inter­actions described above. The overall two-dimensional fingerprint plot is illustrated in Fig. 4 ▸. The Hirshfeld surfaces mapped over *d*_norm_ are shown for the H⋯H, H⋯C/C⋯H, H⋯Br/Br⋯H, H⋯N/N⋯H and H⋯O/O⋯H contacts together with the two-dimensional fingerprint plots associated with their relative contributions to the Hirshfeld surface. At 34.2%, the largest contribution to the overall crystal packing is from H⋯H inter­actions, which are located in the middle region of the fingerprint plot. H⋯C/C⋯H contacts contribute 25.2%, and the H⋯Br/Br⋯H contacts contribute 13.2% to the Hirshfeld surface and both result in a pair of characteristic wings. The H⋯N/N⋯H contacts, represented by a pair of sharp spikes in the fingerprint plot, make a 12.2% contribution to the Hirshfeld surface. Finally, H⋯O/O⋯H contacts, which account for 4.0% of the contribution, are mostly distributed in the middle part of the plot.

## 5. Energy framework analysis

**Figure 5:**
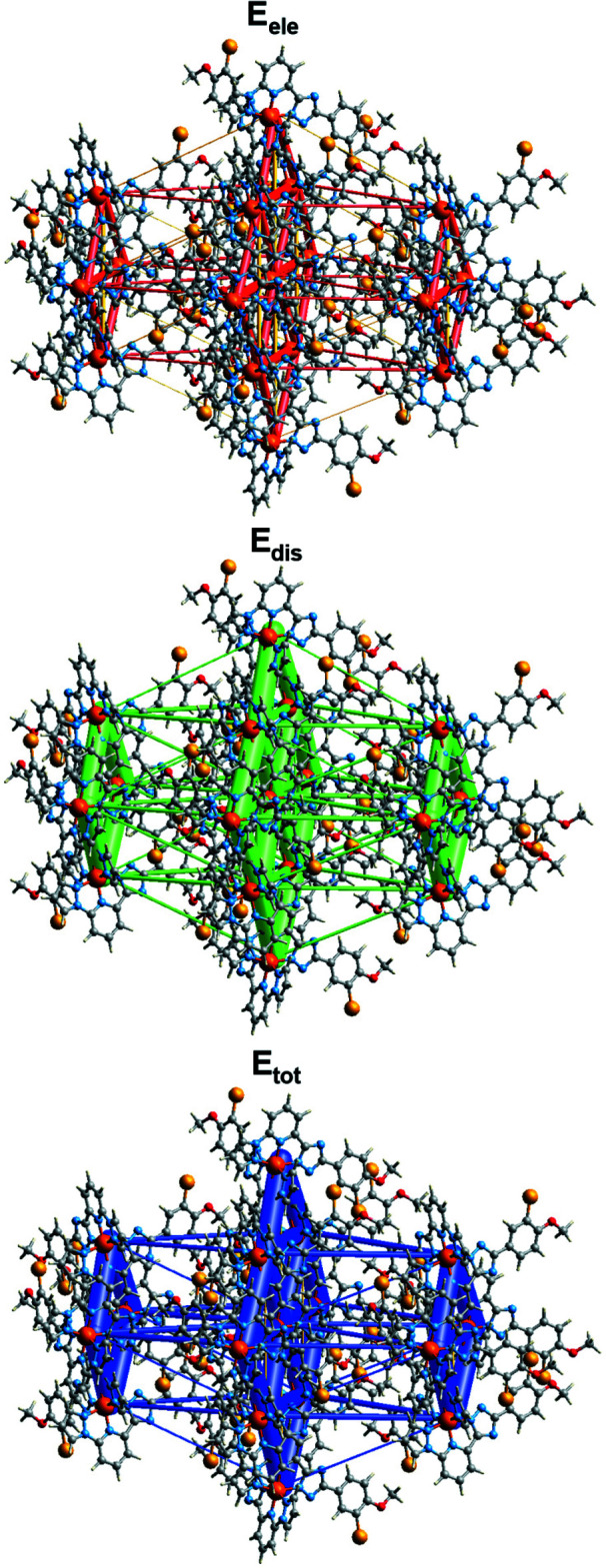
The calculated energy frameworks, showing the electrostatic potential forces (*E*_ele_), dispersion forces (*E*_dis_) and total energy (*E*_tot_) diagrams. Tube size is set at 100 scale, cut-off is 5 kJ mol^−1^.

The energy framework (Spackman *et al.*, 2021 ▸), calculated using the wave function at the HF/3-21G theory level, including the electrostatic potential forces (*E*_ele_), the dispersion forces (*E*_dis_) and the total energy diagrams (*E*_tot_), are shown in Fig. 5 ▸. The cylindrical radii, adjusted to the same scale factor of 100, are proportional to the relative strength of the corresponding energies. The major contribution to the inter­molecular inter­actions is due to the dispersion forces (*E*_dis_), reflecting the dominating inter­actions in the lattice of the neutral asymmetric mol­ecules. The topology of the energy framework resembles the topology of the inter­actions within and between the layers described above. The calculated values *E*_tot_ are in the range 65.2–87.6 kJ mol^−1^ for intra­chain and intra­layer inter­actions, whereas for the inter­layer inter­actions they are within 7.7–23.4 kJ mol^−1^. The colour-coded inter­action mappings within a radius of 3.8 Å of a central reference mol­ecule for the title compound together with full details of the various contributions to the total energy (*E*_tot_) are given in the supporting information.

## 6. Database survey

A search of the Cambridge Structural Database (CSD, Version 5.42, last update February 2021; Groom *et al.*, 2016 ▸) reveals several similar neutral Fe^II^ complexes with a deprotonable azole group, for example, derivatives of a pyrazole-pyridine-tetra­zole (IGERIX and LUTGEO; Gentili *et al.*, 2015 ▸; Senthil Kumar *et al.*, 2015 ▸) and a pyrazole-pyridine-benzimidazole (XODCEB; Shiga *et al.*, 2019 ▸). There are also related complexes based on phenanthroline-tetra­zole, such as QIDJET (Zhang *et al.*, 2007 ▸) and phenanthroline-benzimidazole (DOMQUT; Seredyuk *et al.*, 2014 ▸). Schematic structures of the complexes are shown in Fig. S1 in the supporting information. The Fe—N distances of these complexes in the low-spin state are 1.933–1.959 Å, while in the high-spin state they are in the range 2.179–2.184 Å. The values of the trigonal distortion and CShM(*O_h_*) change correspondingly, and in the low-spin state they are systematically lower than in the high-spin state. Table 2 ▸ collates the structural parameters of the complexes and of the title compound.

## 7. Synthesis and crystallization

The synthesis of the title compound is identical to that reported recently for a similar complex (Seredyuk *et al.*, 2022 ▸). It was produced by layering in a standard test tube. The layering sequence was as follows: the bottom layer contains a solution of [Fe(*L*_2_)](BF_4_)_2_ prepared by dissolving *L* = 2-[5-(3-bromo-4-meth­oxy­phen­yl)-4*H*-1,2,4-triazol-3-yl]-6-(1*H*-pyra­zol-1-yl)pyridine (100 mg, 0.252 mmol) and Fe(BF_4_)_2_·6H_2_O (43 mg, 0.126 mmol) in boiling acetone, to which chloro­form (5 ml) was then added. The middle layer was a methanol–chloro­form mixture (1:10, 10 ml), which was covered by a layer of methanol (10 ml), to which 100 µl of NEt_3_ was added dropwise. The tube was sealed, and black cubic single crystals appeared in 3–4 weeks (yield *ca* 60%). Elemental analysis calculated for C_36_H_32_Br_2_FeN_12_O_4_: C, 47.39; H, 3.54; N, 18.42. Found: C, 47.11; H, 3.74; N, 18.40.

## 8. Refinement

Crystal data, data collection and structure refinement details are summarized in Table 3 ▸. The highest and lowest remaining electron density peaks are located 1.01 and 0.88 Å, respectively, from the Br2 atom. H atoms were refined as riding [C—H = 0.95–0.98 Å with *U*_iso_(H) = 1.2–1.5*U*_eq_(C)]. O-bound H atoms were refined with *U*_iso_(H) = 1.5*U*_eq_(O).

## Supplementary Material

Crystal structure: contains datablock(s) I. DOI: 10.1107/S2056989022010179/dj2053sup1.cif

Structure factors: contains datablock(s) I. DOI: 10.1107/S2056989022010179/dj2053Isup2.hkl

Supporting information file. DOI: 10.1107/S2056989022010179/dj2053Isup4.cdx

Includes energy framework data and schematic structures of similar neutral Fe(II) complexes. DOI: 10.1107/S2056989022010179/dj2053sup3.pdf

CCDC reference: 2215273

Additional supporting information:  crystallographic information; 3D view; checkCIF report

## Figures and Tables

**Table 1:** Geometry (Å, °) of hydrogen bonds and C⋯N interactions in the title compound *Cg*1 and *Cg*2 are the centroids of the C11–C16 and C29–C34 rings, respectively.

*D*—H⋯*A*	*D*—H	H⋯*A*	*D*⋯*A*	*D*—H⋯*A*
C17⋯N6^i^			3.201 (16)	
O3—H3*A*⋯N12	0.84	2.02	2.820 (12)	160
O4—H4⋯N6	0.84	2.06	2.855 (11)	158
C1—H1⋯O4^ii^	0.95	2.22	3.128 (14)	161
C18—H18⋯O3^iii^	0.95	2.27	3.192 (14)	163
C35—H35*A*⋯C30^iv^	0.98	2.62	3.233 (16)	121
C3—H3⋯N5^iii^	0.95	2.45	3.301 (13)	148
C7—H7⋯O4	0.95	2.46	3.310 (11)	148
C22—H22⋯N11^ii^	0.95	2.39	3.317 (13)	166
C20—H20⋯N11^ii^	0.95	2.55	3.389 (13)	148
C5—H5⋯N5^iii^	0.95	2.53	3.440 (12)	161
C17—H17*A*⋯O4^i^	0.98	2.52	3.451 (17)	159
C34—H34⋯C20^v^	0.95	2.63	3.535 (15)	159
C25—H25⋯O3	0.95	2.69	3.542 (13)	150
C18—H18⋯C36^iii^	0.95	2.88	3.65 (2)	138
C2—H2⋯C31^vi^	0.95	2.84	3.639 (15)	143
C2—H2⋯C32^vi^	0.95	2.89	3.656 (15)	139
C2—H2⋯C30^vi^	0.95	2.86	3.734 (11)	154
C2—H2⋯*Cg*2^vi^	0.95	2.57	3.501 (11)	168
C19—H19⋯*Cg*1^vi^	0.95	2.74	3.681 (11)	169

Symmetry codes: (i) 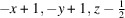 ; (ii) 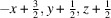 ; (iii) 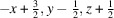 ; (iv) 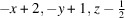 ; (v) 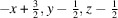 ; (vi) 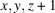 .

**Table 2:** Computed distortion indices (Å,°) for the title compound and similar complexes reported in the literature

CSD refcode	Spin state	<Fe—N>	Σ	Θ	CShM(*O_h_*)
Title compound	LS	1.949	93.3	298.8	2.24
IGERI*X* ^*a*^	HS	2.179	149.7	553.2	6.06
IGERIX01^*a*^	LS	1.986	105.6	350.6	2.85
LUTGEO^*b*^	LS	1.933	85.0	309.6	2.10
XODCEB^*c*^	LS	1.950	87.4	276.6	1.93
DOMQIH^*d*^	LS	1.962	83.8	280.7	2.02
QIDJET01^*e*^	LS	1.970	90.3	341.3	2.47
QIDJET^*e*^	HS	2.184	145.5	553.3	5.88
DOMQUT^*d*^	LS	1.991	88.5	320.0	2.48
DOMQUT02^*d*^	HS	2.183	139.6	486.9	5.31

Notes: (*a*) Gentili *et al.* (2015 ▸); (*b*) Senthil Kumar *et al.* (2015 ▸); (*c*) Shiga *et al.* (2019 ▸); (*d*) Seredyuk *et al.* (2014 ▸); (*e*) Zhang *et al.* (2007 ▸).

**Table 3:** Experimental details

Crystal data	
Chemical formula	[Fe(C_17_H_12_BrN_6_O)_2_]·2CH_4_O
*M*_r_	912.40
Crystal system, space group	Orthorhombic, *P* *n* *a*2_1_
Temperature (K)	180
*a*, *b*, *c* (Å)	27.4318 (10), 12.6723 (4), 10.6434 (3)
*V* (Å^3^)	3699.9 (2)
*Z*	4
Radiation type	Mo *K*α
μ (mm^−1^)	2.63
Crystal size (mm)	0.3 × 0.26 × 0.04

Data collection	
Diffractometer	Xcalibur, Eos
Absorption correction	Multi-scan (*CrysAlis PRO*; Rigaku OD, 2022 ▸)
*T*_min_, *T*_max_	0.772, 1.000
No. of measured, independent and observed [*I* > 2σ(*I*)] reflections	14160, 6227, 4361
*R*_int_	0.061
(sin θ/λ)_max_ (Å^−1^)	0.595

Refinement	
*R*[*F* ^2^ > 2σ(*F* ^2^)], *wR*(*F* ^2^), *S*	0.057, 0.125, 1.03
No. of reflections	6227
No. of parameters	502
No. of restraints	7
H-atom treatment	H-atom parameters constrained
Δρ_max_, Δρ_min_ (e Å^−3^)	1.25, −0.62
Absolute structure	Flack *x* determined using 1444 quotients [(*I* ^+^)−(*I* ^−^)]/[(*I* ^+^)+(*I* ^−^)] (Parsons *et al.*, 2013 ▸)
Absolute structure parameter	−0.009 (8)

Computer programs: *CrysAlis PRO* (Rigaku OD, 2022 ▸), *SHELXT2018/2* (Sheldrick, 2015*a* ▸), *SHELXL2018/3* (Sheldrick, 2015*b* ▸) and *OLEX2* (Dolomanov *et al.*, 2009 ▸).

## supplementary crystallographic information

### Crystal data

**Table:**

[Fe(C_17_H_12_BrN_6_O)_2_]·2CH_4_O	*D*_x_ = 1.638 Mg m^−^^3^
*M**_r_* = 912.40	Mo *K*α radiation, λ = 0.71073 Å
Orthorhombic, *P**n**a*2_1_	Cell parameters from 3167 reflections
*a* = 27.4318 (10) Å	θ = 2.2–25.7°
*b* = 12.6723 (4) Å	µ = 2.63 mm^−^^1^
*c* = 10.6434 (3) Å	*T* = 180 K
*V* = 3699.9 (2) Å^3^	Plate, clear dark red
*Z* = 4	0.3 × 0.26 × 0.04 mm
*F*(000) = 1840	

### Data collection

**Table:**

Xcalibur, Eos diffractometer	6227 independent reflections
Radiation source: fine-focus sealed X-ray tube, Enhance (Mo) X-ray Source	4361 reflections with *I* > 2σ(*I*)
Graphite monochromator	*R*_int_ = 0.061
Detector resolution: 16.1593 pixels mm^-1^	θ_max_ = 25.0°, θ_min_ = 1.8°
ω scans	*h* = −29→32
Absorption correction: multi-scan (CrysAlisPro; Rigaku OD, 2022)	*k* = −15→9
*T*_min_ = 0.772, *T*_max_ = 1.000	*l* = −11→12
14160 measured reflections	

### Refinement

**Table:**

Refinement on *F*^2^	Hydrogen site location: inferred from neighbouring sites
Least-squares matrix: full	H-atom parameters constrained
*R*[*F*^2^ > 2σ(*F*^2^)] = 0.057	*w* = 1/[σ^2^(*F*_o_^2^) + (0.0468*P*)^2^] where *P* = (*F*_o_^2^ + 2*F*_c_^2^)/3
*wR*(*F*^2^) = 0.125	(Δ/σ)_max_ = 0.001
*S* = 1.03	Δρ_max_ = 1.25 e Å^−^^3^
6227 reflections	Δρ_min_ = −0.62 e Å^−^^3^
502 parameters	Absolute structure: Flack *x* determined using 1444 quotients [(*I*^+^)-(*I*^-^)]/[(*I*^+^)+(*I*^-^)] (Parsons *et al.*, 2013)
7 restraints	Absolute structure parameter: −0.009 (8)
Primary atom site location: dual	

### Special details

**Table:**

Geometry. All esds (except the esd in the dihedral angle between two l.s. planes) are estimated using the full covariance matrix. The cell esds are taken into account individually in the estimation of esds in distances, angles and torsion angles; correlations between esds in cell parameters are only used when they are defined by crystal symmetry. An approximate (isotropic) treatment of cell esds is used for estimating esds involving l.s. planes.

### Fractional atomic coordinates and isotropic or equivalent isotropic displacement parameters (Å^2^)

**Table:**

	*x*	*y*	*z*	*U*_iso_*/*U*_eq_	
Br1	0.93947 (6)	0.18179 (10)	0.18738 (16)	0.0755 (6)	
Br2	0.58117 (5)	0.86423 (9)	0.16575 (14)	0.0533 (4)	
Fe1	0.74880 (4)	0.51357 (9)	0.66488 (14)	0.0165 (3)	
O1	0.9850 (3)	0.3512 (6)	0.0344 (7)	0.046 (2)	
O2	0.5033 (3)	0.7175 (7)	0.0845 (8)	0.059 (3)	
N1	0.7925 (3)	0.4642 (7)	0.7986 (7)	0.021 (2)	
N2	0.7854 (3)	0.3616 (6)	0.8310 (6)	0.0190 (19)	
N3	0.7331 (2)	0.3663 (5)	0.6688 (8)	0.0169 (16)	
N4	0.6980 (3)	0.5090 (7)	0.5358 (7)	0.019 (2)	
N5	0.6735 (3)	0.5777 (6)	0.4591 (7)	0.021 (2)	
N6	0.6424 (3)	0.4144 (6)	0.4290 (7)	0.021 (2)	
N7	0.7030 (3)	0.5599 (6)	0.7956 (7)	0.0179 (19)	
N8	0.7095 (3)	0.6637 (6)	0.8297 (7)	0.0203 (19)	
N9	0.7639 (2)	0.6605 (5)	0.6733 (7)	0.0157 (16)	
N10	0.8015 (3)	0.5208 (7)	0.5376 (7)	0.0167 (19)	
N11	0.8252 (3)	0.4538 (7)	0.4581 (7)	0.020 (2)	
N12	0.8561 (3)	0.6197 (6)	0.4353 (7)	0.020 (2)	
C1	0.8232 (4)	0.5020 (9)	0.8838 (9)	0.022 (3)	
H1	0.836027	0.571689	0.884392	0.027*	
C2	0.8342 (4)	0.4225 (8)	0.9744 (9)	0.027 (3)	
H2	0.854203	0.429649	1.046766	0.032*	
C3	0.8103 (3)	0.3351 (8)	0.9362 (9)	0.022 (2)	
H3	0.810827	0.267895	0.975646	0.027*	
C4	0.7534 (3)	0.3030 (8)	0.7529 (8)	0.020 (2)	
C5	0.7444 (3)	0.1970 (8)	0.7604 (8)	0.020 (2)	
H5	0.760428	0.153348	0.819944	0.024*	
C6	0.7104 (3)	0.1564 (7)	0.6761 (9)	0.026 (2)	
H6	0.703580	0.082904	0.675727	0.032*	
C7	0.6865 (3)	0.2219 (8)	0.5934 (8)	0.023 (2)	
H7	0.662303	0.194369	0.538543	0.028*	
C8	0.6979 (3)	0.3278 (8)	0.5907 (8)	0.018 (2)	
C9	0.6790 (3)	0.4128 (8)	0.5154 (7)	0.016 (2)	
C10	0.6407 (4)	0.5181 (8)	0.3985 (8)	0.020 (2)	
C11	0.6061 (3)	0.5658 (8)	0.3117 (8)	0.021 (2)	
C12	0.5688 (4)	0.5087 (9)	0.2563 (10)	0.042 (3)	
H12	0.566690	0.435012	0.271727	0.051*	
C13	0.5339 (4)	0.5569 (9)	0.1775 (13)	0.049 (3)	
H13	0.508661	0.515389	0.141389	0.059*	
C14	0.5360 (4)	0.6632 (9)	0.1525 (11)	0.034 (3)	
C15	0.5737 (3)	0.7203 (9)	0.2037 (9)	0.029 (3)	
C16	0.6080 (4)	0.6728 (8)	0.2824 (9)	0.026 (3)	
H16	0.633427	0.714686	0.316951	0.031*	
C17	0.4597 (5)	0.6610 (12)	0.0437 (14)	0.085 (6)	
H17A	0.436960	0.710675	0.004257	0.128*	
H17B	0.443970	0.628133	0.116611	0.128*	
H17C	0.468752	0.606305	−0.017004	0.128*	
C18	0.6699 (4)	0.5218 (9)	0.8754 (9)	0.027 (3)	
H18	0.656975	0.452322	0.872907	0.033*	
C19	0.6568 (4)	0.6007 (9)	0.9651 (9)	0.028 (3)	
H19	0.634576	0.593789	1.033020	0.033*	
C20	0.6829 (4)	0.6878 (8)	0.9327 (9)	0.022 (2)	
H20	0.682436	0.753910	0.974832	0.026*	
C21	0.7426 (3)	0.7227 (8)	0.7576 (8)	0.019 (2)	
C22	0.7510 (4)	0.8292 (8)	0.7670 (9)	0.024 (2)	
H22	0.734218	0.871698	0.826391	0.029*	
C23	0.7855 (3)	0.8719 (7)	0.6848 (9)	0.028 (2)	
H23	0.792262	0.945451	0.686681	0.033*	
C25	0.8098 (4)	0.8074 (8)	0.6009 (9)	0.024 (2)	
H25	0.833605	0.836305	0.545977	0.029*	
C26	0.7993 (3)	0.7007 (8)	0.5971 (8)	0.015 (2)	
C27	0.8201 (3)	0.6167 (8)	0.5207 (8)	0.017 (2)	
C28	0.8572 (3)	0.5168 (9)	0.3993 (8)	0.022 (2)	
C29	0.8908 (3)	0.4756 (8)	0.3034 (9)	0.021 (2)	
C30	0.9141 (4)	0.5434 (9)	0.2193 (8)	0.028 (3)	
H30	0.908122	0.617120	0.223076	0.034*	
C31	0.9464 (4)	0.5030 (10)	0.1295 (9)	0.029 (3)	
H31	0.962898	0.550064	0.074511	0.034*	
C32	0.9544 (4)	0.3969 (10)	0.1196 (9)	0.034 (3)	
C33	0.9309 (4)	0.3287 (9)	0.2023 (10)	0.037 (3)	
C34	0.8993 (4)	0.3676 (9)	0.2928 (9)	0.029 (3)	
H34	0.883359	0.320139	0.348246	0.035*	
C35	1.0089 (5)	0.4170 (10)	−0.0556 (11)	0.062 (4)	
H35A	1.026381	0.372877	−0.116380	0.093*	
H35B	1.032148	0.463288	−0.012460	0.093*	
H35C	0.984662	0.460036	−0.099718	0.093*	
O3	0.8946 (3)	0.8132 (7)	0.3501 (8)	0.054 (2)	
H3A	0.889770	0.749515	0.368323	0.080*	
C36	0.9355 (6)	0.8495 (13)	0.4134 (18)	0.101 (6)	
H36A	0.964669	0.816116	0.378324	0.151*	
H36B	0.932720	0.831820	0.502734	0.151*	
H36C	0.937869	0.926235	0.403763	0.151*	
O4	0.6173 (3)	0.2092 (6)	0.3391 (7)	0.040 (2)	
H4	0.620155	0.274742	0.348513	0.059*	
C24	0.5796 (4)	0.1885 (10)	0.2562 (11)	0.054 (4)	
H24A	0.581928	0.115298	0.227028	0.082*	
H24B	0.581953	0.236323	0.184082	0.082*	
H24C	0.548314	0.199154	0.298680	0.082*	

### Atomic displacement parameters (Å^2^)

**Table:**

	*U*^11^	*U*^22^	*U*^33^	*U*^12^	*U*^13^	*U*^23^
Br1	0.0982 (11)	0.0278 (8)	0.1007 (13)	0.0159 (8)	0.0583 (11)	−0.0030 (9)
Br2	0.0659 (8)	0.0299 (7)	0.0640 (8)	0.0033 (6)	−0.0228 (9)	0.0170 (8)
Fe1	0.0203 (6)	0.0113 (7)	0.0179 (6)	−0.0009 (6)	−0.0005 (7)	0.0006 (8)
O1	0.048 (5)	0.041 (6)	0.050 (5)	0.001 (4)	0.030 (4)	−0.010 (4)
O2	0.048 (5)	0.050 (6)	0.077 (6)	−0.010 (5)	−0.044 (4)	0.030 (5)
N1	0.023 (5)	0.016 (5)	0.023 (5)	−0.008 (4)	0.003 (4)	0.001 (4)
N2	0.028 (5)	0.011 (5)	0.019 (4)	−0.001 (4)	0.007 (3)	0.007 (4)
N3	0.027 (4)	0.011 (4)	0.013 (4)	−0.003 (3)	0.008 (4)	0.004 (4)
N4	0.028 (5)	0.015 (5)	0.013 (4)	0.001 (4)	0.000 (3)	−0.001 (4)
N5	0.021 (5)	0.010 (5)	0.031 (5)	−0.005 (4)	−0.001 (4)	0.009 (4)
N6	0.025 (5)	0.015 (5)	0.022 (5)	−0.002 (4)	−0.004 (4)	0.000 (4)
N7	0.024 (5)	0.010 (5)	0.020 (4)	−0.002 (4)	0.008 (4)	0.004 (4)
N8	0.030 (5)	0.009 (5)	0.022 (5)	0.002 (4)	0.001 (4)	−0.007 (4)
N9	0.026 (4)	0.009 (4)	0.013 (4)	−0.001 (3)	−0.008 (4)	−0.002 (4)
N10	0.015 (4)	0.016 (5)	0.018 (4)	0.000 (4)	−0.004 (3)	0.002 (4)
N11	0.027 (5)	0.014 (5)	0.017 (5)	−0.005 (4)	0.005 (4)	−0.010 (4)
N12	0.024 (5)	0.010 (5)	0.026 (5)	−0.004 (4)	0.004 (4)	−0.003 (4)
C1	0.019 (6)	0.013 (6)	0.034 (6)	−0.003 (5)	−0.003 (5)	−0.002 (5)
C2	0.033 (6)	0.022 (7)	0.025 (6)	0.008 (6)	−0.006 (5)	0.001 (5)
C3	0.029 (6)	0.018 (6)	0.020 (5)	0.011 (5)	−0.005 (4)	0.005 (4)
C4	0.025 (6)	0.018 (6)	0.015 (5)	−0.001 (5)	−0.004 (4)	0.001 (4)
C5	0.027 (6)	0.013 (6)	0.020 (5)	0.003 (5)	0.003 (4)	0.007 (4)
C6	0.034 (5)	0.020 (5)	0.026 (6)	−0.005 (5)	0.007 (6)	0.001 (6)
C7	0.028 (6)	0.024 (7)	0.018 (5)	0.000 (5)	−0.005 (4)	−0.005 (5)
C8	0.018 (5)	0.013 (6)	0.022 (5)	−0.003 (5)	0.004 (4)	−0.006 (4)
C9	0.021 (6)	0.016 (6)	0.009 (5)	0.001 (5)	−0.002 (4)	0.000 (4)
C10	0.025 (6)	0.018 (7)	0.018 (5)	0.001 (5)	−0.002 (4)	0.003 (4)
C11	0.022 (6)	0.021 (6)	0.021 (5)	−0.005 (5)	−0.002 (4)	0.003 (5)
C12	0.039 (8)	0.023 (7)	0.064 (8)	−0.007 (6)	−0.007 (6)	0.015 (6)
C13	0.039 (6)	0.042 (8)	0.066 (8)	−0.014 (6)	−0.033 (7)	0.012 (8)
C14	0.026 (5)	0.041 (7)	0.035 (6)	−0.002 (5)	−0.009 (5)	0.021 (6)
C15	0.026 (6)	0.031 (7)	0.028 (6)	0.001 (5)	−0.002 (5)	0.013 (5)
C16	0.026 (6)	0.021 (6)	0.031 (6)	−0.003 (5)	−0.007 (5)	−0.001 (5)
C17	0.069 (10)	0.081 (12)	0.105 (11)	−0.027 (9)	−0.065 (9)	0.032 (9)
C18	0.031 (7)	0.018 (7)	0.032 (6)	0.000 (6)	0.001 (5)	0.004 (5)
C19	0.039 (7)	0.028 (7)	0.017 (5)	0.006 (6)	0.009 (5)	0.000 (5)
C20	0.027 (6)	0.019 (6)	0.019 (5)	0.009 (5)	0.007 (4)	0.003 (5)
C21	0.022 (6)	0.015 (6)	0.019 (5)	−0.005 (5)	−0.006 (4)	0.003 (4)
C22	0.026 (6)	0.023 (6)	0.023 (5)	0.001 (5)	−0.005 (4)	−0.005 (5)
C23	0.046 (6)	0.009 (5)	0.028 (6)	−0.002 (5)	−0.001 (6)	−0.002 (5)
C25	0.030 (6)	0.018 (6)	0.023 (5)	−0.007 (5)	0.001 (4)	0.005 (5)
C26	0.013 (5)	0.014 (6)	0.020 (5)	−0.002 (5)	−0.002 (4)	0.002 (4)
C27	0.021 (5)	0.021 (6)	0.010 (5)	−0.007 (5)	0.003 (4)	−0.001 (4)
C28	0.016 (5)	0.025 (7)	0.023 (6)	0.002 (5)	0.000 (4)	0.002 (5)
C29	0.018 (5)	0.025 (7)	0.020 (5)	0.005 (5)	0.000 (4)	−0.004 (5)
C30	0.030 (6)	0.030 (7)	0.026 (5)	−0.008 (6)	0.004 (5)	−0.011 (5)
C31	0.020 (6)	0.038 (8)	0.028 (6)	−0.007 (6)	0.003 (4)	−0.001 (5)
C32	0.033 (7)	0.041 (8)	0.028 (6)	0.010 (6)	0.001 (5)	−0.005 (5)
C33	0.050 (7)	0.028 (7)	0.033 (7)	0.007 (6)	0.004 (6)	−0.008 (5)
C34	0.025 (6)	0.031 (7)	0.032 (6)	−0.004 (6)	0.012 (5)	−0.001 (5)
C35	0.072 (10)	0.057 (11)	0.056 (8)	−0.007 (9)	0.038 (8)	−0.005 (8)
O3	0.067 (6)	0.023 (5)	0.070 (6)	−0.009 (5)	0.009 (5)	0.013 (4)
C36	0.058 (11)	0.065 (13)	0.179 (18)	−0.023 (10)	−0.015 (12)	0.025 (12)
O4	0.044 (5)	0.021 (5)	0.053 (5)	0.003 (4)	−0.018 (4)	−0.012 (4)
C24	0.060 (9)	0.047 (10)	0.056 (9)	0.012 (8)	−0.019 (7)	−0.022 (7)

### Geometric parameters (Å, º)

**Table:**

Br1—C33	1.883 (11)	C11—C12	1.384 (14)
Br2—C15	1.879 (11)	C11—C16	1.392 (13)
Fe1—N1	1.964 (8)	C12—H12	0.9500
Fe1—N3	1.916 (7)	C12—C13	1.412 (14)
Fe1—N4	1.958 (8)	C13—H13	0.9500
Fe1—N7	1.964 (8)	C13—C14	1.374 (14)
Fe1—N9	1.909 (6)	C14—C15	1.375 (13)
Fe1—N10	1.982 (8)	C15—C16	1.396 (13)
O1—C32	1.364 (12)	C16—H16	0.9500
O1—C35	1.430 (13)	C17—H17A	0.9800
O2—C14	1.342 (11)	C17—H17B	0.9800
O2—C17	1.462 (14)	C17—H17C	0.9800
N1—N2	1.359 (10)	C18—H18	0.9500
N1—C1	1.328 (12)	C18—C19	1.429 (14)
N2—C3	1.354 (11)	C19—H19	0.9500
N2—C4	1.418 (12)	C19—C20	1.360 (14)
N3—C4	1.324 (11)	C20—H20	0.9500
N3—C8	1.363 (11)	C21—C22	1.373 (13)
N4—N5	1.370 (11)	C22—H22	0.9500
N4—C9	1.344 (12)	C22—C23	1.398 (13)
N5—C10	1.340 (12)	C23—H23	0.9500
N6—C9	1.361 (11)	C23—C25	1.382 (13)
N6—C10	1.354 (12)	C25—H25	0.9500
N7—N8	1.376 (10)	C25—C26	1.384 (13)
N7—C18	1.334 (12)	C26—C27	1.455 (13)
N8—C20	1.352 (11)	C28—C29	1.472 (13)
N8—C21	1.404 (11)	C29—C30	1.395 (14)
N9—C21	1.329 (11)	C29—C34	1.393 (13)
N9—C26	1.365 (11)	C30—H30	0.9500
N10—N11	1.364 (11)	C30—C31	1.399 (12)
N10—C27	1.331 (12)	C31—H31	0.9500
N11—C28	1.341 (12)	C31—C32	1.367 (15)
N12—C27	1.341 (11)	C32—C33	1.393 (15)
N12—C28	1.359 (12)	C33—C34	1.385 (14)
C1—H1	0.9500	C34—H34	0.9500
C1—C2	1.426 (13)	C35—H35A	0.9800
C2—H2	0.9500	C35—H35B	0.9800
C2—C3	1.350 (14)	C35—H35C	0.9800
C3—H3	0.9500	O3—H3A	0.8400
C4—C5	1.369 (12)	O3—C36	1.387 (16)
C5—H5	0.9500	C36—H36A	0.9800
C5—C6	1.392 (13)	C36—H36B	0.9800
C6—H6	0.9500	C36—H36C	0.9800
C6—C7	1.376 (13)	O4—H4	0.8400
C7—H7	0.9500	O4—C24	1.384 (12)
C7—C8	1.379 (13)	C24—H24A	0.9800
C8—C9	1.440 (13)	C24—H24B	0.9800
C10—C11	1.456 (13)	C24—H24C	0.9800
			
N1—Fe1—N7	88.4 (3)	O2—C14—C13	125.4 (10)
N1—Fe1—N10	93.7 (3)	O2—C14—C15	116.5 (10)
N3—Fe1—N1	79.1 (3)	C13—C14—C15	118.1 (10)
N3—Fe1—N4	80.0 (3)	C14—C15—Br2	120.5 (7)
N3—Fe1—N7	97.6 (3)	C14—C15—C16	121.2 (10)
N3—Fe1—N10	102.9 (3)	C16—C15—Br2	118.3 (8)
N4—Fe1—N1	159.1 (4)	C11—C16—C15	121.9 (9)
N4—Fe1—N7	93.0 (3)	C11—C16—H16	119.1
N4—Fe1—N10	92.3 (3)	C15—C16—H16	119.1
N7—Fe1—N10	159.4 (3)	O2—C17—H17A	109.5
N9—Fe1—N1	98.3 (3)	O2—C17—H17B	109.5
N9—Fe1—N3	176.0 (4)	O2—C17—H17C	109.5
N9—Fe1—N4	102.5 (3)	H17A—C17—H17B	109.5
N9—Fe1—N7	79.3 (3)	H17A—C17—H17C	109.5
N9—Fe1—N10	80.2 (3)	H17B—C17—H17C	109.5
C32—O1—C35	118.7 (10)	N7—C18—H18	125.0
C14—O2—C17	117.1 (10)	N7—C18—C19	110.1 (10)
N2—N1—Fe1	113.6 (6)	C19—C18—H18	125.0
C1—N1—Fe1	140.1 (8)	C18—C19—H19	127.3
C1—N1—N2	105.3 (8)	C20—C19—C18	105.4 (9)
N1—N2—C4	116.2 (7)	C20—C19—H19	127.3
C3—N2—N1	111.9 (8)	N8—C20—C19	107.8 (9)
C3—N2—C4	131.9 (8)	N8—C20—H20	126.1
C4—N3—Fe1	120.6 (6)	C19—C20—H20	126.1
C4—N3—C8	119.6 (8)	N9—C21—N8	109.7 (8)
C8—N3—Fe1	119.6 (6)	N9—C21—C22	123.9 (9)
N5—N4—Fe1	138.5 (7)	C22—C21—N8	126.3 (9)
C9—N4—Fe1	114.6 (6)	C21—C22—H22	121.6
C9—N4—N5	106.8 (8)	C21—C22—C23	116.7 (9)
C10—N5—N4	105.0 (8)	C23—C22—H22	121.6
C10—N6—C9	101.7 (8)	C22—C23—H23	120.0
N8—N7—Fe1	113.0 (6)	C25—C23—C22	120.1 (9)
C18—N7—Fe1	141.1 (7)	C25—C23—H23	120.0
C18—N7—N8	105.5 (8)	C23—C25—H25	120.1
N7—N8—C21	116.6 (7)	C23—C25—C26	119.8 (9)
C20—N8—N7	111.1 (8)	C26—C25—H25	120.1
C20—N8—C21	132.2 (8)	N9—C26—C25	119.7 (8)
C21—N9—Fe1	121.0 (6)	N9—C26—C27	109.8 (8)
C21—N9—C26	119.5 (8)	C25—C26—C27	130.5 (9)
C26—N9—Fe1	119.4 (6)	N10—C27—N12	113.6 (9)
N11—N10—Fe1	138.0 (7)	N10—C27—C26	116.2 (8)
C27—N10—Fe1	114.5 (6)	N12—C27—C26	130.3 (9)
C27—N10—N11	107.5 (8)	N11—C28—N12	115.1 (9)
C28—N11—N10	103.3 (8)	N11—C28—C29	121.5 (10)
C27—N12—C28	100.4 (8)	N12—C28—C29	123.3 (9)
N1—C1—H1	125.1	C30—C29—C28	121.0 (10)
N1—C1—C2	109.9 (10)	C34—C29—C28	120.6 (9)
C2—C1—H1	125.1	C34—C29—C30	118.4 (9)
C1—C2—H2	127.1	C29—C30—H30	119.9
C3—C2—C1	105.9 (9)	C29—C30—C31	120.2 (11)
C3—C2—H2	127.1	C31—C30—H30	119.9
N2—C3—H3	126.5	C30—C31—H31	119.5
C2—C3—N2	106.9 (9)	C32—C31—C30	120.9 (10)
C2—C3—H3	126.5	C32—C31—H31	119.5
N3—C4—N2	109.9 (8)	O1—C32—C31	124.6 (10)
N3—C4—C5	123.8 (8)	O1—C32—C33	116.2 (11)
C5—C4—N2	126.3 (8)	C31—C32—C33	119.2 (10)
C4—C5—H5	121.7	C32—C33—Br1	120.2 (8)
C4—C5—C6	116.5 (9)	C34—C33—Br1	119.3 (9)
C6—C5—H5	121.7	C34—C33—C32	120.5 (11)
C5—C6—H6	119.7	C29—C34—H34	119.6
C7—C6—C5	120.6 (9)	C33—C34—C29	120.7 (10)
C7—C6—H6	119.7	C33—C34—H34	119.6
C6—C7—H7	120.3	O1—C35—H35A	109.5
C6—C7—C8	119.5 (9)	O1—C35—H35B	109.5
C8—C7—H7	120.3	O1—C35—H35C	109.5
N3—C8—C7	119.8 (9)	H35A—C35—H35B	109.5
N3—C8—C9	109.1 (8)	H35A—C35—H35C	109.5
C7—C8—C9	131.1 (9)	H35B—C35—H35C	109.5
N4—C9—N6	112.4 (8)	C36—O3—H3A	109.5
N4—C9—C8	116.6 (8)	O3—C36—H36A	109.5
N6—C9—C8	130.8 (9)	O3—C36—H36B	109.5
N5—C10—N6	114.0 (9)	O3—C36—H36C	109.5
N5—C10—C11	120.6 (9)	H36A—C36—H36B	109.5
N6—C10—C11	125.3 (9)	H36A—C36—H36C	109.5
C12—C11—C10	122.3 (10)	H36B—C36—H36C	109.5
C12—C11—C16	116.3 (9)	C24—O4—H4	109.5
C16—C11—C10	121.4 (9)	O4—C24—H24A	109.5
C11—C12—H12	119.1	O4—C24—H24B	109.5
C11—C12—C13	121.9 (10)	O4—C24—H24C	109.5
C13—C12—H12	119.1	H24A—C24—H24B	109.5
C12—C13—H13	119.7	H24A—C24—H24C	109.5
C14—C13—C12	120.6 (10)	H24B—C24—H24C	109.5
C14—C13—H13	119.7		
			
Br1—C33—C34—C29	−177.7 (8)	C3—N2—C4—N3	172.4 (9)
Br2—C15—C16—C11	−177.8 (7)	C3—N2—C4—C5	−8.0 (16)
Fe1—N1—N2—C3	−169.9 (6)	C4—N2—C3—C2	−178.2 (9)
Fe1—N1—N2—C4	8.8 (10)	C4—N3—C8—C7	−4.9 (13)
Fe1—N1—C1—C2	165.1 (8)	C4—N3—C8—C9	175.6 (8)
Fe1—N3—C4—N2	0.4 (10)	C4—C5—C6—C7	−2.0 (13)
Fe1—N3—C4—C5	−179.2 (7)	C5—C6—C7—C8	2.7 (14)
Fe1—N3—C8—C7	−179.9 (7)	C6—C7—C8—N3	0.7 (14)
Fe1—N3—C8—C9	0.5 (10)	C6—C7—C8—C9	−179.8 (9)
Fe1—N4—N5—C10	−176.2 (7)	C7—C8—C9—N4	179.9 (9)
Fe1—N4—C9—N6	177.2 (6)	C7—C8—C9—N6	3.8 (17)
Fe1—N4—C9—C8	0.5 (10)	C8—N3—C4—N2	−174.6 (7)
Fe1—N7—N8—C20	−170.3 (6)	C8—N3—C4—C5	5.7 (14)
Fe1—N7—N8—C21	7.5 (9)	C9—N4—N5—C10	0.7 (10)
Fe1—N7—C18—C19	167.7 (8)	C9—N6—C10—N5	0.4 (11)
Fe1—N9—C21—N8	−1.0 (10)	C9—N6—C10—C11	−177.5 (9)
Fe1—N9—C21—C22	−178.6 (7)	C10—N6—C9—N4	0.1 (10)
Fe1—N9—C26—C25	178.7 (7)	C10—N6—C9—C8	176.3 (10)
Fe1—N9—C26—C27	−0.7 (10)	C10—C11—C12—C13	176.6 (10)
Fe1—N10—N11—C28	180.0 (7)	C10—C11—C16—C15	−177.1 (9)
Fe1—N10—C27—N12	179.3 (6)	C11—C12—C13—C14	0.4 (19)
Fe1—N10—C27—C26	−0.1 (10)	C12—C11—C16—C15	1.3 (15)
O1—C32—C33—Br1	−3.1 (13)	C12—C13—C14—O2	−176.3 (11)
O1—C32—C33—C34	179.6 (10)	C12—C13—C14—C15	1.6 (19)
O2—C14—C15—Br2	−5.6 (14)	C13—C14—C15—Br2	176.3 (9)
O2—C14—C15—C16	176.0 (10)	C13—C14—C15—C16	−2.1 (17)
N1—N2—C3—C2	0.3 (11)	C14—C15—C16—C11	0.6 (16)
N1—N2—C4—N3	−6.1 (11)	C16—C11—C12—C13	−1.9 (16)
N1—N2—C4—C5	173.6 (9)	C17—O2—C14—C13	6.0 (19)
N1—C1—C2—C3	2.4 (12)	C17—O2—C14—C15	−171.9 (11)
N2—N1—C1—C2	−2.2 (11)	C18—N7—N8—C20	3.1 (10)
N2—C4—C5—C6	178.2 (8)	C18—N7—N8—C21	−179.0 (8)
N3—C4—C5—C6	−2.2 (14)	C18—C19—C20—N8	0.6 (12)
N3—C8—C9—N4	−0.6 (11)	C20—N8—C21—N9	172.9 (9)
N3—C8—C9—N6	−176.7 (8)	C20—N8—C21—C22	−9.5 (16)
N4—N5—C10—N6	−0.7 (11)	C21—N8—C20—C19	−179.8 (9)
N4—N5—C10—C11	177.2 (8)	C21—N9—C26—C25	−5.5 (12)
N5—N4—C9—N6	−0.5 (10)	C21—N9—C26—C27	175.1 (8)
N5—N4—C9—C8	−177.3 (8)	C21—C22—C23—C25	−1.0 (14)
N5—C10—C11—C12	−173.9 (9)	C22—C23—C25—C26	1.0 (14)
N5—C10—C11—C16	4.5 (15)	C23—C25—C26—N9	2.3 (13)
N6—C10—C11—C12	3.9 (15)	C23—C25—C26—C27	−178.5 (9)
N6—C10—C11—C16	−177.7 (9)	C25—C26—C27—N10	−178.9 (9)
N7—N8—C20—C19	−2.3 (11)	C25—C26—C27—N12	1.9 (17)
N7—N8—C21—N9	−4.4 (11)	C26—N9—C21—N8	−176.6 (7)
N7—N8—C21—C22	173.2 (9)	C26—N9—C21—C22	5.7 (14)
N7—C18—C19—C20	1.4 (12)	C27—N10—N11—C28	0.4 (10)
N8—N7—C18—C19	−2.7 (11)	C27—N12—C28—N11	−0.9 (11)
N8—C21—C22—C23	−179.6 (8)	C27—N12—C28—C29	179.4 (8)
N9—C21—C22—C23	−2.4 (14)	C28—N12—C27—N10	1.1 (10)
N9—C26—C27—N10	0.4 (11)	C28—N12—C27—C26	−179.6 (9)
N9—C26—C27—N12	−178.8 (9)	C28—C29—C30—C31	179.4 (8)
N10—N11—C28—N12	0.3 (11)	C28—C29—C34—C33	179.8 (9)
N10—N11—C28—C29	−180.0 (8)	C29—C30—C31—C32	2.1 (15)
N11—N10—C27—N12	−1.0 (10)	C30—C29—C34—C33	1.1 (15)
N11—N10—C27—C26	179.6 (8)	C30—C31—C32—O1	179.6 (9)
N11—C28—C29—C30	162.3 (9)	C30—C31—C32—C33	−1.4 (15)
N11—C28—C29—C34	−16.3 (14)	C31—C32—C33—Br1	177.8 (8)
N12—C28—C29—C30	−18.0 (15)	C31—C32—C33—C34	0.6 (16)
N12—C28—C29—C34	163.4 (9)	C32—C33—C34—C29	−0.4 (16)
C1—N1—N2—C3	1.2 (10)	C34—C29—C30—C31	−1.9 (14)
C1—N1—N2—C4	180.0 (8)	C35—O1—C32—C31	−3.0 (16)
C1—C2—C3—N2	−1.6 (11)	C35—O1—C32—C33	178.0 (10)

### Hydrogen-bond geometry (Å, º)

*Cg*1 and *Cg*2 are the centroids of the C11–C16 and C29–C34 rings, respectively.

**Table:**

*D*—H···*A*	*D*—H	H···*A*	*D*···*A*	*D*—H···*A*
C17···N6^i^			3.201 (16)	
O3—H3*A*···N12	0.84	2.02	2.820 (12)	160
O4—H4···N6	0.84	2.06	2.855 (11)	158
C1—H1···O4^ii^	0.95	2.22	3.128 (14)	161
C18—H18···O3^iii^	0.95	2.27	3.192 (14)	163
C35—H35*A*···C30^iv^	0.98	2.62	3.233 (16)	121
C3—H3···N5^iii^	0.95	2.45	3.301 (13)	148
C7—H7···O4	0.95	2.46	3.310 (11)	148
C22—H22···N11^ii^	0.95	2.39	3.317 (13)	166
C20—H20···N11^ii^	0.95	2.55	3.389 (13)	148
C5—H5···N5^iii^	0.95	2.53	3.440 (12)	161
C17—H17*A*···O4^i^	0.98	2.52	3.451 (17)	159
C34—H34···C20^v^	0.95	2.63	3.535 (15)	159
C25—H25···O3	0.95	2.69	3.542 (13)	150
C18—H18···C36^iii^	0.95	2.88	3.65 (2)	138
C2—H2···C31^vi^	0.95	2.84	3.639 (15)	143
C2—H2···C32^vi^	0.95	2.89	3.656 (15)	139
C2—H2···C30^vi^	0.95	2.86	3.734 (11)	154
C2—H2···*Cg*2^vi^	0.95	2.57	3.501 (11)	168
C19—H19···*Cg*1^vi^	0.95	2.74	3.681 (11)	169

Symmetry codes: (i) −*x*+1, −*y*+1, *z*−1/2; (ii) −*x*+3/2, *y*+1/2, *z*+1/2; (iii) −*x*+3/2, *y*−1/2, *z*+1/2; (iv) −*x*+2, −*y*+1, *z*−1/2; (v) −*x*+3/2, *y*−1/2, *z*−1/2; (vi) *x*, *y*, *z*+1.

### Geometry (Å, °) of hydrogen bonds and C···N interactions in the title compound.

**Table:**

*D*–H···*A*	*D*–H	*D*···*A*	H···*A*	*D*–H···*A*	Symmetry operation
C17···N6	-	3.20 (2)	-	-	1-x,1-y,-1/2+z
O3-H···N12	0.84 (1)	2.82 (1)	2.02 (1)	159.5 (5)	x,y,z
O4-H···N6	0.84 (1)	2.86 (1)	2.06 (2)	158.0 (5)	x,y,z
C1-H···O4	0.95 (1)	3.13 (1)	2.22 (1)	160.6 (5)	1.5-x,1/2+y,1/2+z
C18-H···O3	0.95 (1)	3.19 (1)	2.27 (1)	162.6 (5)	1.5-x,-1/2+y,1/2+z
C35-H···C30	0.95 (1)	3.23 (2)	2.62 (2)	121.0 (5)	2-x,1-y,-1/2+z
C3-H···N5	0.95 (1)	3.30 (1)	2.46 (2)	148.4 (5)	1.5-x,-1/2+y,1/2+z
C7-H···O4	0.95 (1)	3.31 (1)	2.46 (1)	148.4 (5)	x,y,z
C22-H···N11	0.95 (1)	3.32 (1)	2.39 (1)	165.6 (5)	1.5-x,1/2+y,1/2+z
C20-H···N11	0.95 (1)	3.39 (1)	2.55 (2)	147.6 (5)	1.5-x,1/2+y,1/2+z
C5-H···N5	0.95 (1)	3.44 (1)	2.53 (2)	160.7 (5)	1.5-x,-1/2+y,1/2+z
C17-H···O4	0.95 (1)	3.45 (2)	2.52 (2)	159.1 (5)	1-x,1-y,-1/2+z
C34-H···C20	0.95 (1)	3.53 (2)	2.63 (1)	159.1 (5)	1.5-x,-1/2+y,-1/2+z
C25-H···O3	0.95 (1)	3.54 (1)	2.69 (1)	149.6 (5)	x,y,z
C18-H···C36	0.95 (1)	3.65 (2)	2.88 (1)	138.0 (5)	1.5-x,-1/2+y,1/2+z
C2–H···C31	0.95 (1)	3.64 (2)	2.84 (1)	143.0 (5)	x,y,1+z
C2-H···C32	0.95 (1)	3.66 (2)	2.89 (2)	139.0 (5)	x,y,1+z
C2–H···C30	0.95 (1)	3.73 (1)	2.86 (1)	154.4 (5)	x,y,1+z
