# Supplementary material for: Crystal structure of bis­{3-(3-bromo-4-methoxyphenyl)-5-[6-(1H-pyrazol-1-yl)pyridin-2-yl]-1,2,4-triazol-3-ato}­iron(II) methano­l disolvate
Source: Acta Crystallogr E Crystallogr Commun. 2022 Oct 28;78(Pt 11):1138–42. doi: 10.1107/S2056989022010179 (PMC9638973; doi:10.1107/S2056989022010179)
Supplement: Supplementary file 4 [file e-78-01138-sup3.pdf]

# Supporting file S1

## Energy framework analysis

The full colour-coded interaction mappings of a central reference molecule for the title compound. Full details of the various contributions to the total energy ($E_{tot}$) are also given.

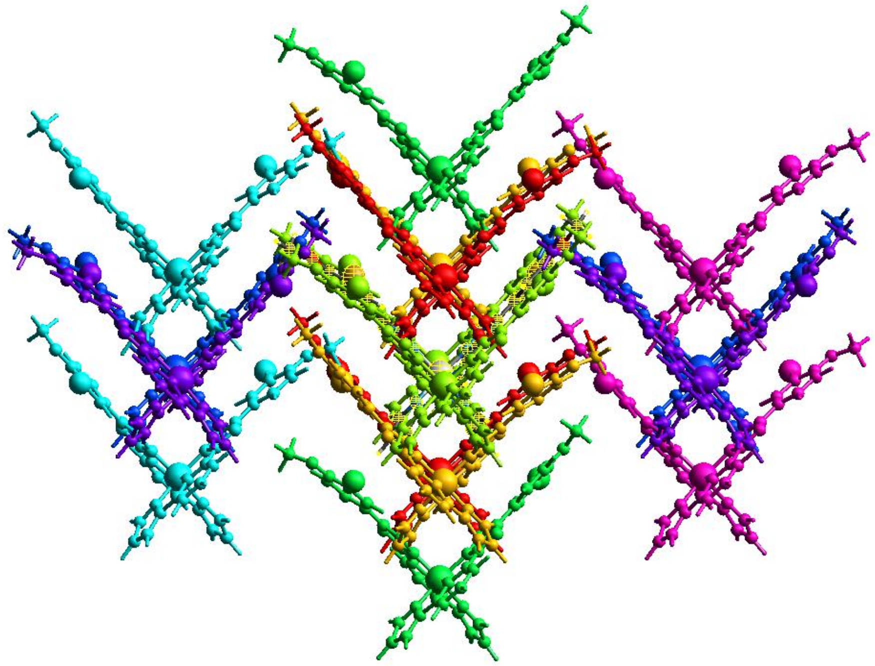

| | N | Symop | R | $E_{ele}$ | $E_{pol}$ | $E_{dis}$ | $E_{rep}$ | $E_{tot}$ |
|---|---|---|---|---|---|---|---|---|
| (red) | 2 | -x+1/2, y+1/2, z+1/2 | 8.27 | -33.8 | -27.9 | -100.6 | 68.7 | -87.6 |
| (orange) | 2 | -x+1/2, y+1/2, z+1/2 | 8.27 | -32.7 | -28.9 | -91.6 | 61.9 | -84.6 |
| (yellow-green) | 2 | x, y, z | 12.67 | 15.4 | -3.0 | -7.3 | 0.8 | 7.9 |
| (green) | 2 | x, y, z | 10.64 | -38.2 | -10.2 | -38.7 | 18.9 | -65.2 |
| (cyan) | 2 | -x, -y, z+1/2 | 14.70 | -10.9 | -4.8 | -18.0 | 8.6 | -23.4 |
| (blue) | 2 | x+1/2, -y+1/2, z | 15.32 | 3.5 | -1.2 | -7.9 | 2.5 | -2.3 |
| (purple) | 2 | x+1/2, -y+1/2, z | 14.91 | 2.3 | -2.8 | -13.3 | 4.5 | -7.7 |
| (magenta) | 2 | -x, -y, z+1/2 | 14.74 | -9.3 | -3.9 | -20.3 | 15.8 | -17.5 |

**Figure S1.** Schematic structure of neutral $Fe^{II}$ complexes with deprotonable azol-based ligands.

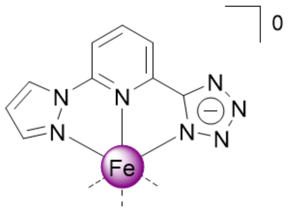

**IGERIX**

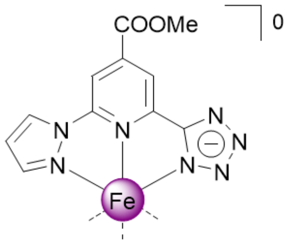

**LUTGEO**

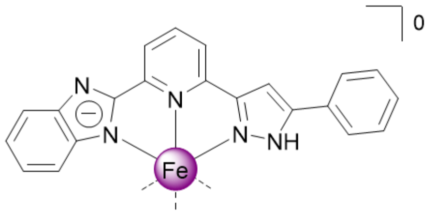

**XODCEB**

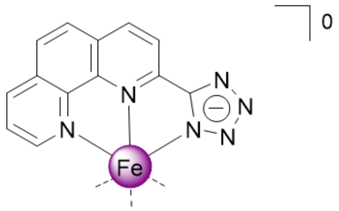

**QIDJET**

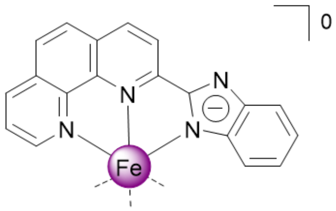

**DOMQIH**

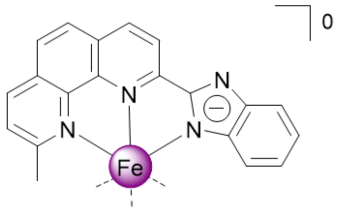

**DOMQUT**
